# Evaluation of Version 4 of the Emergency Severity Index in US Emergency Departments for the Rate of Mistriage

**DOI:** 10.1001/jamanetworkopen.2023.3404

**Published:** 2023-03-17

**Authors:** Dana R. Sax, E. Margaret Warton, Dustin G. Mark, David R. Vinson, Mamata V. Kene, Dustin W. Ballard, Tina J. Vitale, Katherine R. McGaughey, Aaron Beardsley, Jesse M. Pines, Mary E. Reed

**Affiliations:** 1 Department of Emergency Medicine, Kaiser Permanente Oakland Medical Center, Oakland, California; 2 Division of Research, Kaiser Permanente Northern California, Oakland; 3 Department of Emergency Medicine, Kaiser Permanente Roseville Medical Center, Roseville, California; 4 Department of Emergency Medicine, Kaiser Permanente San Leandro Medical Center, San Leandro, California; 5 Department of Emergency Medicine, Kaiser Permanente San Rafael Medical Center, San Rafael, California; 6 US Acute Care Solutions, Arlington, Virginia

## Abstract

**Importance:**

Accurate emergency department (ED) triage is essential to prioritize the most critically ill patients and distribute resources appropriately. The most used triage system in the US is the Emergency Severity Index (ESI).

**Objectives:**

To derive and validate an algorithm to assess the rate of mistriage and to identify characteristics associated with mistriage.

**Design, Setting, and Participants:**

This retrospective cohort study created operational definitions for each ESI level that use ED visit electronic health record data to classify encounters as undertriaged, overtriaged, or correctly triaged. These definitions were applied to a retrospective cohort to assess variation in triage accuracy by facility and patient characteristics in 21 EDs within the Kaiser Permanente Northern California (KPNC) health care system. All ED encounters by patients 18 years and older between January 1, 2016, and December 31, 2020, were assessed for eligibility. During that time, version 4 of the ESI was in use by these EDs. Encounters with missing ESI or incomplete ED time variables and patients who left against medical advice or without being seen were excluded. Data were analyzed between January 1, 2021, and November 30, 2022.

**Exposures:**

Assigned ESI level.

**Main Outcomes and Measures:**

Rate of undertriage and overtriage by assigned ESI level based on a mistriage algorithm and patient and visit characteristics associated with undertriage and overtriage.

**Results:**

A total of 5 315 176 ED encounters were included. The mean (SD) patient age was 52 (21) years; 44.3% of patients were men and 55.7% were women. In terms of race and ethnicity, 11.1% of participants were Asian, 15.1% were Black, 21.4% were Hispanic, 44.0% were non-Hispanic White, and 8.5% were of other (includes American Indian or Alaska Native, Native Hawaiian or other Pacific Islander, and multiple races or ethnicities), unknown, or missing race or ethnicity. Mistriage occurred in 1 713 260 encounters (32.2%), of which 176 131 (3.3%) were undertriaged and 1 537 129 (28.9%) were overtriaged. The sensitivity of ESI to identify a patient with high-acuity illness (correctly assigning ESI I or II among patients who had a life-stabilizing intervention) was 65.9%. In adjusted analyses, Black patients had a 4.6% (95% CI, 4.3%-4.9%) greater relative risk of overtriage and an 18.5% (95% CI, 16.9%-20.0%) greater relative risk of undertriage compared with White patients, while Black male patients had a 9.9% (95% CI, 9.8%-10.0%) greater relative risk of overtriage and a 41.0% (95% CI, 40.0%-41.9%) greater relative risk of undertriage compared with White female patients. High relative risk of undertriage was found among patients taking high-risk medications (30.3% [95% CI, 28.3%-32.4%]) and those with a greater comorbidity burden (22.4% [95% CI, 20.1%-24.4%]) and recent intensive care unit utilization (36.7% [95% CI, 30.5%-41.4%]).

**Conclusions and Relevance:**

In this retrospective cohort study of over 5 million ED encounters, mistriage with version 4 of the ESI was common. Quality improvement should focus on limiting critical undertriage, optimizing resource allocation by patient need, and promoting equity. Future research should include assessments based on version 5 of the ESI, which was released after this study was completed.

## Key Points

**Question:**

What is the rate of emergency department (ED) mistriage, and what patient and visit characteristics are associated with mistriage?

**Findings:**

In a cohort study of 5 315 176 adult ED encounters across 21 hospitals using version 4 of the Emergency Severity Index assessed between January 1, 2016, and December 31, 2020, mistriage occurred in an estimated one-third of encounters, and the sensitivity to identify a critically ill patient was 66%. Key patient sociodemographic and clinical characteristics were associated with mistriage.

**Meaning:**

These findings suggest that development of more data-driven, standardized triage processes may limit critical undertriage, optimize resource allocation, and promote more equitable care in EDs.

## Teaser

This cohort study derives and validates an electronic health record–based tool to assess the frequency of Emergency Severity Index (ESI) mistriage and assesses patient and visit characteristics associated with mistriage in the emergency department.

## Introduction

When demand for emergency department (ED) resources (eg, beds and staff) exceeds supply, patients must wait for care. Wait times have lengthened as ED visits and intensity of care (eg, number of tests, imaging, and medications ordered) have increased.^1^ This has contributed to crowding, which negatively impacts quality and outcomes.^2,3,4,5,6^ Prolonged waits are associated with higher risks of mortality, hospital admission, 30-day readmission, patient dissatisfaction, and costs.^7,8^

Emergency department triage, or the sorting of patients based on predicted acuity and resource needs, is necessary to ensure patients who require immediate care are treated first. The triage system used in over 70% of EDs across the US,^9^ the Emergency Severity Index (ESI), was developed in 1998.^10^ The index was originally maintained by the Agency for Healthcare Research and Quality. In 2019, the Emergency Nurses Association acquired the ESI and subsequently released version 4 of the ESI Implementation Handbook in 2020^11^ and version 5 of the ESI and ESI Implementation Handbook in 2023.^12^ The ESI uses an algorithm to categorize patients from level I, the most critically ill, to level V, the least critically ill and resource intensive. ESI version 4 brought changes to criteria for ESI Level I to better classify patients needing immediate, life-saving interventions as well as updates to the pediatric fever criteria.^13^ ESI version 5 and version 5 of the ESI Implementation Handbook added assessment tools, clarifying language to algorithm branchpoints, a look into how bias and stigmas could lead to inaccurate triage decisions, and a new course examination to assess how nurses apply ESI.^12^

The assignment of ESI relies on a combination of assessment based on initial vital signs and triage nurse judgment. The latter can introduce error, bias,^14,15,16^ and variation in triage assignments and reduce interrater reliability.^17,18,19,20,21,22,23,24^ Studies have also found limited validity, defined as how close an assigned acuity is to true acuity.^19,25,26,27^ Undertriage, or failure to identify patients with acutely severe illness (eg, sepsis, trauma, and myocardial ischemia) from those with less urgent needs, contributes to delays in care and increased morbidity and mortality.^28,29,30,31,32^ By contrast, overtriage, or the overestimation of patient acuity or resource needs, may be associated with resource overuse.^33^ Several studies conducted prior to ESI version 5 have shown an overrepresentation of ESI III assignments and poor differentiation of patient acuity,^34,35^ which can contribute to ED crowding and worse outcomes.^2,3,4,5,6^ Herein we use the term *mistriage* to encompass both undertriage and overtriage.

Several studies have evaluated the accuracy of ED triage systems, including ESI version 4. Many are limited by the lack of a criterion standard to define mistriage.^36,37^ A systematic review found over 75% of studies used expert opinion based on manual medical record review or case simulations, with small sample sizes limiting generalizability.^25^ To our knowledge, no studies have used detailed data from the electronic health record (EHR) to assess the rate of ED mistriage. A validated operational definition of mistriage may be helpful for quality improvement in the ED triage process and as a potential quality measure to assess nurse performance.

We aimed to derive and validate an EHR-based tool to assess the frequency of mistriage with ESI version 4 and assess patient and visit characteristics associated with mistriage. We used version 4 because that version was in use by EDs within our system during the time of our study (2016-2020). We hypothesized that mistriage rates would vary considerably, and specific patient and visit characteristics would be associated with higher risk of mistriage.

## Methods

### Study Design

We first created operational definitions that use EHR data to measure mistriage. Specifically, we developed separate definitions of undertriage and overtriage by ESI level (version 4) using expert analysis and a modified Delphi technique. We then applied these definitions to a large data set to assess variation by facility and patient characteristics. The study was approved by the Kaiser Permanente Northern California (KPNC) institutional review board. Informed consent was not necessary as this was a data-only retrospective analysis. The study followed the Strengthening the Reporting of Observational Studies in Epidemiology (STROBE) reporting guideline. 

### Study Setting and Population

We conducted a retrospective cohort study among all patients 18 years or older who had an ED visit between January 1, 2016, and December 31, 2020, across the 21 EDs in KPNC. Kaiser Permanente Northern California is an integrated health care delivery system that provides comprehensive medical care for more than 4.5 million patients with approximately 1.2 million ED visits per year. At that time, version 4 of the ESI was used at all EDs in this study. We excluded encounters with missing ESI, incomplete ED time variables, and those who left the ED against medical advice or without being seen by a clinician. Members of KPNC include approximately 33% of the population in areas served and are representative of the demographic and socioeconomic diversity of the surrounding and statewide population.^38,39^

The 21 hospital-based EDs included in this study are community EDs with annual volumes ranging from 28 000 to 130 000 encounters per year. The mean (SD) hospital and intensive care unit (ICU) admission rates across all facilities were 12.3% (0.3%) and 1.3% (0.1%), respectively.

### Variables

The ESI algorithm is subjective in its assignment of high-risk situations and resource use. Our study team of 5 emergency physicians (D.R.S., D.G.M., D.R.V., D.W.B., and M.V.K.) and 3 emergency nurses (T.J.V., K.R.M., and A.B.) sought to develop an objective system to measure critical illness that together with resource utilization could be used to determine triage accuracy.

To differentiate among varying levels of clinical acuity, we used a modified Delphi technique^40^ to develop lists of key ED interventions, including medications, procedures, and care processes readily available in the EHR. We categorized interventions using a hierarchical schema from level 4 (least critical) to level 1 (most critical). We used the most critical event during a patient’s ED stay to define a patient’s intervention level. We then used these intervention levels as well as ED resource counts to define correct triage vs undertriage or overtriage for each ESI level. Resource use counts were based on the standard ESI resource definition, which counts the number of different types of resources. We used time stamps to define each critical intervention to allow for dynamic changes in patient condition after arrival.

We collected patient and visit data as variables that might be associated with mistriage, including demographic information from EHR databases and neighborhood socioeconomic status at the census block group level. Members of the KPNC health plan were defined as having active KPNC health plan membership for at least 9 of the past 12 months. We collected prior ED, inpatient, and ICU utilization from the EHR. Specific medications that might put a patient at higher risk of mistriage were pulled from pharmacy databases. We ascertained information on coexisting illnesses based on history of diagnoses using *International Statistical Classification of Diseases and Related Health Problems, Tenth Revision* codes. For each patient, we obtained an internally derived and validated comorbidity risk score (Comorbidity Point Score, version 2 [COPS2]).^41^ COPS2 is calculated quarterly and requires at least 1 month of KPNC health plan membership during the quarter in which it is calculated. To develop our definitions of mistriage, we collected the timing of key ED events and processes, including procedures, specialty consultation, medications dispensed, and ED disposition (discharge, admission, or transfer). Race and ethnicity data (Asian, Black, Hispanic, non-Hispanic White, other [including American Indian or Alaska Native, Native Hawaiian or other Pacific Islander, and multiple races or ethnicities], unknown, or missing) were collected from the EHR to assess possible disparities in triage accuracy. We collected visit time, date, facility, and study year to assess for variation in triage accuracy by time or location.

### Data Validation

Study coinvestigators performed several rounds of manual medical record review to ensure (1) accurate capture of all components of the mistriage algorithm and (2) that reviewers agreed with the algorithm’s assessment. This process was continued iteratively until we reached over 95% accuracy of event capture and over 90% agreement with the algorithm’s assessment. More than 400 records were reviewed to reach these accuracy and agreement levels. The final definitions of undertriage and overtriage for each ESI level are reported in Table 1 and eTable 1 in Supplement 1.

**Table 1.** Novel Definitions of Undertriage and Overtriage for Each ESI Level (Version 4)

Assigned ESI level	Clinical outcomes
**Occurrence of clinical outcomes indicates undertriage**	
V	Any resource used^a^; any hospital admission; any level 1, 2, 3, or 4 intervention occurred^b^
IV	>1 Type resource used; any level 1, 2, 3, or 4 intervention occurred; admission to hospital (any level of care)
III	Any level 1 or 2 intervention occurred
II	Any level 1 intervention occurred
**Occurrence of clinical outcomes indicates overtriage**	
I	<2 Resources used and no level 1 or 2 intervention occurred
II and III	<2 Resources used
IV	No resources used

Abbreviations: ED, emergency department; ESI, Emergency Severity Index; ICU, intensive care unit.

^a^ Resource use was defined as in version 4 of the ESI, and each different type of resource is counted as a resource, not the individual tests or imaging studies. Resources include laboratory analysis, electrocardiography, radiography, computed tomography, magnetic resonance imaging, diagnostic ultrasonography (not point of care), angiography, intravenous fluids, and intravenous, intramuscular, or nebulized medications. Oral medications, tetanus immunizations, point-of-care testing, history and physical examination, saline or heparin lock, prescription refills, simple wound care, crutches, splints, and slings do not count. In our electronic health record, specialty consultation and simple procedures (laceration repair, Foley catheter) were not consistently available as discreet fields, so these were not counted as resources used.

^b^ Level 1 intervention indicates lifesaving intervention within 1 hour of ED arrival, including invasive ventilation; tier I critical medications; admission to ICU, operating room, catheterization unit, or other hospital; transfusion; and/or death in ED; level 2, any level 1 intervention beyond the first hour of ED arrival, tier II critical medication, parenteral psychotropic medication, noninvasive ventilation, or intraosseous line; level 3, admission to ICU or operating room or transfer to other hospital, critical procedures, tier III critical medication, and/or no level 1 or 2 intervention; and level 4, tier IV medication, transfusion beyond the first hour, and/or no level 1, 2, or 3 intervention. A complete description of each intervention level is found in eTable 1 in Supplement 1.

We then applied the algorithm to a random set of medical records to validate the definitions with reviewers blinded to the algorithm’s assessment. Each encounter was reviewed by 2 coinvestigators (each of the 8 clinicians reviewed medical records, and each medical record was reviewed by random pairs of clinicians). A total of 200 medical records were reviewed, and the agreement level at this phase was 96%. Cases of disagreement between algorithm and reviewer assessment of mistriage were due to complexity that was harder to capture electronically, including level of pain, prolonged consultations, and psychosocial challenges.

### Outcomes

We applied the algorithm to the full study cohort to compare algorithm-determined ESI (version 4) levels against the ED triage-assigned ESI levels. We compared baseline patient and visit characteristics by correct triage, undertriage, and overtriage. We further defined meaningful undertriage as ESI III or IV encounters with level 1 or 2 interventions or ESI V encounters with level 1 to 4 interventions or at least 2 resources used. We defined meaningful overtriage as ESI II or III encounters that required zero resources prior to ED discharge.

### Statistical Analysis

Data were analyzed between January 1, 2021, and November 30, 2022. We assessed variation in mean frequency of undertriage and overtriage and time to initial ESI assignment across centers using least squares means with adjustment for multiple comparisons (Tukey-Kramer test).^42^ We used median imputation for continuous variables if there was any missingness and added a flag to indicate observations with imputed values. We included these flags in the models if they were statistically significant. For categorical variables with greater than 0.5% missingness, we created a separate missing category.

We used log binomial models to estimate the relative risks of undertriage compared with correct triage and overtriage compared with correct triage for multiple patient- and visit-level characteristics. We performed a sensitivity analysis of the multivariate models excluding non-KPNC health plan members to assess the impact of missing data among nonmembers on triage accuracy and the direction of variable coefficients. All analyses were performed using SAS, version 9.4 (SAS Institute Inc). Two-sided α < .05 indicated statistical significance.

## Results

We included 5 315 176 ED encounters over the study period. The mean (SD) age of study participants was 52 (21) years; 55.7% were women and 44.3% were men. In terms of race and ethnicity, 11.1% of participants were Asian, 15.1% were Black, 21.4% were Hispanic, 44.0% were non-Hispanic White, and 8.5% were of other (American Indian or Alaska Native, Native Hawaiian or other Pacific Islander, or multiple races or ethnicities), unknown, or missing race or ethnicity. Only 33.6% of encounters occurred during office hours, defined as an arrival Monday to Friday, 9 am to 5 pm. A total of 80.6% of encounters were made by KPNC members. The lowest ED volumes were in 2020, likely due to decreases in ED utilization seen nationally at the beginning of the COVID-19 pandemic.^43^ Cohort assembly is shown in the eFigure in Supplement 1.

Overall, 3 262 047 encounters (61.4%) were assigned a midlevel triage category (ESI III), while 33 491 (0.6%) were ESI I, 929 555 (18.1%) were ESI II, 1 046 806 (19.7%) were ESI IV, and 43 277 (0.8%) were ESI V. Applying study-developed definitions, we estimate that mistriage occurred in 1 713 260 ED encounters (32.2%). Undertriage and overtriage occurred in 176 131 (3.3%) and 1 537 129 (28.9%) cases, respectively. The Figure shows there were 57 794 encounters with meaningful undertriage (32.8% of all undertriaged encounters), and 373 958 encounters with meaningful overtriage (24.3% of all overtriaged encounters). The greatest proportion of undertriaged encounters were ESI III encounters that should have been ESI II, largely driven by 24 181 undertriaged ESI III encounters (4.4%) in which a tier II medication was used. The most common tier II medication used was dextrose 50% (accounting for 11 984 undertriaged encounters with tier II medication use [49.5%]), and 6359 (54.6%) of these patients were using insulin or a sulfonylurea. Table 2 presents patient and visit characteristics of the full cohort and separately among patients who were correctly triaged, overtriaged, and undertriaged. Facility-level rates of undertriage and overtriage varied 3.0-fold and 2.5-fold, respectively, and mean time to initial triage assignment varied 4.0-fold.

**Figure.**
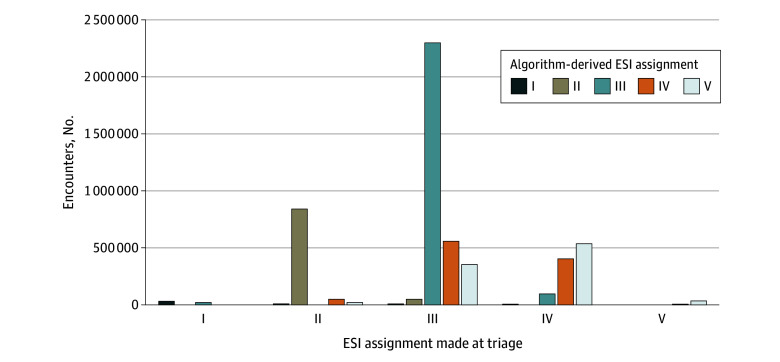
Assigned Emergency Severity Index (ESI), Version 4, Compared With Algorithm-Derived ESI The ESI is a 5-level emergency department triage algorithm that sorts patients into 5 groups from I (most urgent) to V (least urgent). Comparison assumes exact resource and critical care needs were known at triage. Meaningful undertriage was defined as ESI III or IV encounters with level 1 or 2 interventions (ie, should have been ESI I or II, respectively) or ESI V encounters with level 1 to 4 interventions or 2 or more resources used. Meaningful overtriage was defined as ESI II or III encounters that required zero resources prior to ED discharge (ie, should have been ESI V).

**Table 2.** Patient Characteristics of Full Study Cohort and by Triage Group

Characteristic	Participant group, No. (%)^a^			
	Overall (N = 5 315 176)	Correct triage (n = 3 601 916)	Overtriage (n = 1 537 129)	Undertriage (n = 176 131)
Age category, y				
18-29	1 015 662 (19.1)	564 716 (15.7)	417 492 (27.2)	33 454 (19.0)
30-39	815 214 (15.3)	492 144 (13.7)	297 843 (19.4)	25 227 (14.3)
40-49	707 641 (13.3)	464 538 (12.9)	220 648 (14.4)	22 455 (12.7)
50-59	785 815 (14.8)	546 290 (15.2)	212 631 (13.8)	26 894 (15.3)
60-69	733 716 (13.8)	540 933 (15.0)	165 871 (10.8)	26 912 (15.3)
70-79	622 107 (11.7)	482 695 (13.4)	117 363 (7.6)	22 049 (12.5)
≥80	635 021 (11.9)	510 600 (14.2)	105 281 (6.8)	19 140 (10.9)
Sex				
Female	2 962 867 (55.7)	2 035 993 (56.5)	838 211 (54.5)	88 663 (50.3)
Male	2 352 309 (44.3)	1 565 923 (43.5)	698 918 (45.5)	87 468 (49.7)
Race and ethnicity				
Asian	590 576 (11.1)	407 941 (11.3)	164 240 (10.7)	18 395 (10.4)
Black	800 984 (15.1)	491 746 (13.7)	277 160 (18.0)	32 078 (18.2)
Hispanic	1 137 459 (21.4)	747 455 (20.8)	352 281 (22.9)	37 723 (21.4)
Non-Hispanic White	2 336 058 (44.0)	1 655 750 (46.0)	607 392 (39.5)	72 916 (41.4)
Unknown, missing, or other^b^	450 099 (8.5)	299 024 (8.3)	136 056 (8.9)	15 019 (8.5)
English is primary language	4 868 004 (91.6)	3 294 538 (91.5)	1 411 936 (91.9)	161 530 (91.7)
Standardized NDI, mean (SD)^c^	0.06 (0.97)	0.02 (0.96)	0.14 (0.99)	0.12 (0.98)
Encounter during office hours^d^	1 787 363 (33.6)	1 260 714 (35.0)	469 277 (30.5)	57 372 (32.6)
Patient arrived by ambulance	933 633 (17.6)	751 999 (20.9)	142 897 (9.3)	38 737 (22.0)
KPNC member at encounter date	4 281 599 (80.6)	3 012 831 (83.6)	1 130 722 (73.6)	138 046 (78.4)
High-risk medications^e^				
Immunosuppression	64 523 (1.2)	50 433 (1.4)	11 657 (0.8)	2433 (1.4)
Chemotherapy	197 575 (3.7)	155 889 (4.3)	35 535 (2.3)	6151 (3.5)
Insulin or sulfonylurea	533 872 (10.0)	416 288 (11.6)	92 687 (6.0)	24 897 (14.1)
Anticoagulation	345 323 (6.5)	281 291 (7.8)	50 759 (3.3)	13 273 (7.5)
COPS2 risk level^f^				
Low (<20)	2 655 487 (50.0)	1 724 218 (47.9)	851 057 (55.4)	80 212 (45.5)
Medium (20 to <65)	1 976 329 (37.2)	1 319 655 (36.6)	590 549 (38.4)	66 125 (37.5)
High (≥65)	683 360 (12.9)	558 043 (15.5)	95 523 (6.2)	29 794 (16.9)
Recent health care utilization (past 30 d)				
Any hospitalization	217 835 (4.1)	175 472 (4.9)	33 897 (2.2)	8466 (4.8)
Any intensive care	41 512 (0.8)	33 946 (0.9)	5200 (0.3)	2366 (1.3)
Any ED encounter	734 264 (13.8)	517 756 (14.4)	192 580 (12.5)	23 928 (13.6)
Study year				
2016	1 015 732 (19.1)	668 405 (18.6)	316 188 (20.6)	31 139 (17.7)
2017	1 068 197 (20.1)	712 669 (19.8)	320 395 (20.8)	35 133 (19.9)
2018	1 108 704 (20.9)	750 359 (20.8)	320 516 (20.9)	37 829 (21.5)
2019	1 155 638 (21.7)	785 820 (21.8)	331 598 (21.6)	38 220 (21.7)
2020	966 905 (18.2)	684 663 (19.0)	248 432 (16.2)	33 810 (19.2)

Abbreviations: COPS2, Comorbidity Point Score, version 2; ED, emergency department; KPNC, Kaiser Permanente Northern California; NDI, Neighborhood Deprivation Index.

^a^ Percentages have been rounded and might not total 100. Only 2 variables had missingness greater than 0.5%. All patient and visit characteristic differences by triage category (correct, overtriage, or undertriage) were significant with *P* < .001. Percentages for age, race, and COPS2 represent column percentages within each respective type of characteristic (age, race, or COPS2). Other percentages represent the absolute percent of encounters with these variables within each row and column.

^b^ Includes American Indian or Alaska Native, Native Hawaiian or other Pacific Islander, and more than 1 race.

^c^ The NDI for each census tract is based on 13 socioeconomic measures. Scores range from −3.6 to 2.8, with higher values indicating more neighborhood deprivation (lower socioeconomic status). The missingness rate for NDI was 0.8%.

^d^ Office hours were defined as ED arrival times from 9 am to 5 pm, Monday through Friday. Nonoffice hours were defined as ED arrival times outside of these time windows.

^e^ Active treatment with chemotherapy, immunosuppressants, anticoagulants, and insulin or sulfonylureas was defined as a medication dispensed within the past 6 months.

^f^ Indicates a longitudinal comorbidity score based on 12 months of patient data.^41^ The missingness rate for COPS2 was 18.2%, which is similar to that for non-KPNC membership (19.4%). Nonmembers generally do not have COPS2 scores because the score’s calculation relies on membership for at least 1 month in the quarter the score is calculated.

Two variables had greater than 0.5% missingness: Neighborhood Deprivation Index (missingness rate of 0.8%) and COPS2 (missingness rate of 18.3%). Among the 18.3% without a COPS2 score, 90.3% did not have KPNC health plan membership.

eTables 2 and 3 in Supplement 1 display the frequency of resource utilization and levels 1 to 4 interventions by ESI level. The sensitivity of ESI was 50.0%, specificity was 96.8%, positive predictive value was 16.6, and negative predictive value was 0.5 for patients with low-acuity and low-resource needs (correctly assigning ESI IV or V among patients who used <2 resources and had no critical interventions). The sensitivity of ESI was 65.9%, specificity was 83.4%, positive predictive value was 3.3, and negative predictive value was 0.4 for patients with high-acuity needs (correctly assigning ESI I or II among patients with a level 1 or 2 intervention). Among the most critically ill patients (those with a level 1 intervention), 17 292 (60.9%) were undertriaged (13 278 [46.8%] were assigned ESI II and 4014 [14.1%] were assigned ESI III-V).

### Multivariate Analysis of Variables Associated With Mistriage

In multivariate adjusted analyses, we identified multiple patient sociodemographic characteristics associated with undertriage and overtriage (Table 3). Younger, male, and Black patients, patients living in poorer neighborhoods, and non-KPNC members were significantly more likely to be mistriaged in both directions (undertriage and overtriage) compared with older, female, and White patients, patients living in less poor neighborhoods, and KPNC members. Black patients had a 4.6% (95% CI, 4.3%-4.9%) greater relative risk of overtriage and an 18.5% (95% CI, 16.9%-20.0%) greater relative risk of undertriage compared with White patients, while Black male patients had a 9.9% (95% CI, 9.8%-10.0%) higher relative risk of overtriage and a 41.0% (95% CI, 40.0%-41.9%) higher relative risk of undertriage compared with White female patients.

**Table 3.** Multivariate Adjusted Relative Risks of Patient and Visit Characteristics Associated With Undertriage and Overtriage

Characteristic	Relative risk (95% CI)^a^	
	Undertriage	Overtriage
Demographic		
Age, y		
18-29	1 [Reference]	1 [Reference]
30-39	0.89 (0.87-0.90)	0.92 (0.92-0.92)
40-49	0.84 (0.83-0.86)	0.83 (0.82-0.84)
50-59	0.84 (0.82-0.85)	0.77 (0.77-0.88)
60-69	0.83 (0.82-0.85)	0.73 (0.72-0.73)
70-79	0.77 (0.76-0.79)	0.70 (0.69-0.71)
≥80	0.66 (0.65-0.68)	0.70 (0.69-0.70)
Women		
No	1 [Reference]	1 [Reference]
Yes	0.81 (0.80-0.82)	0.94 (0.95-0.95)
Race and ethnicity		
Non-Hispanic White	1 [Reference]	1 [Reference]
Asian	1.01 (0.99-1.02)	0.98 (0.97-0.98)
Black	1.18 (1.17-1.20)	1.05 (1.04-1.05)
Hispanic	1.01 (0.99-1.02)	0.99 (0.98-0.99)
Unknown, missing, or other^b^	1.05 (0.99-1.02)	1.01 (1.01-1.02)
Non-English primary language		
No	1 [Reference]	1 [Reference]
Yes	0.95 (0.93-0.96)	0.96 (0.95-0.96)
NDI (standardized)		
1-SD increase	1.03 (1.02-1.03)	1.01 (1.01-1.01)
NDI is imputed^c^		
No	1 [Reference]	1 [Reference]
Yes	1.03 (1.02-1.03)	0.98 (0.97-0.99)
Visit measures		
Visit during office hours^d^		
No	1 [Reference]	1 [Reference]
Yes	0.92 (0.91-0.93)	0.93 (0.92-0.93)
Arrived by ambulance		
No	1 [Reference]	1 [Reference]
Yes	1.09 (1.08-1.10)	0.68 (0.67-0.68)
Year of visit		
2016	1 [Reference]	1 [Reference]
2017	1.06 (1.04-1.07)	0.98 (0.96-0.98)
2018	1.08 (1.07-1.10)	0.96 (0.96-0.96)
2019	1.05 (1.04-1.06)	0.95 (0.95-0.96)
2020	1.07 (1.05-1.08)	0.89 (0.89-0.89)
Non-KPNC health plan member at visit		
No	1 [Reference]	1 [Reference]
Yes	1.15 (1.12-1.18)	1.10 (1.09-1.10)
Risk factors^e^		
Immunosuppression		
No	1 [Reference]	1 [Reference]
2019	0.99 (0.05-1.03)	0.90 (0.90-0.91)
Chemotherapy		
No	1 [Reference]	1 [Reference]
2020	0.86 (0.84-0.88)	0.90 (0.90-0.91)
Insulin or sulfonylurea use		
No	1 [Reference]	1 [Reference]
Yes	1.30 (1.28-1.32)	0.90 (0.90-0.91)
Anticoagulation use		
No	1 [Reference]	1 [Reference]
Yes	1.03 (1.01-1.05)	0.90 (0.89-0.90)
COPS2 comorbidity score		
Low (<20)	1 [Reference]	1 [Reference]
High (≥65)	1.22 (1.20-1.24)	0.83 (0.83-0.84)
Moderate (20 to <65)	0.94 (0.93-0.95)	0.91 (0.90-0.91)
COPS2 score imputed^f^		
No	1 [Reference]	1 [Reference]
Yes	1.15 (1.12-1.18)	1.13 (1.13-1.14)
Utilization in prior 30 d		
Hospitalized		
No	1 [Reference]	1 [Reference]
Yes	1.01 (0.99-1.03)	0.85 (0.84-0.85)
ICU admission		
No	1 [Reference]	1 [Reference]
Yes	1.30 (1.30-1.41)	0.96 (0.94-0.97)
ED visit in prior 30 d		
No	1 [Reference]	1 [Reference]
Yes	0.86 (0.85-0.87)	1.04 (1.04-1.04)

Abbreviations: COPS2, Comorbidity Point Score, version 2; ED, emergency department; ICU, intensive care unit; KPNC, Kaiser Permanente Northern California; NDI, Neighborhood Deprivation Index.

^a^ Relative risks less than 1.00 indicate lower relative risk of undertriage or overtriage; greater than 1.00, higher relative risk of undertriage or overtriage; and 95% CIs that cross 1, no significant change. The model was also adjusted for study facility and triage vital signs (heart rate, blood pressure, respiratory rate, temperature, and pulse oximetry).

^b^ Includes American Indian or Alaska Native, Native Hawaiian or other Pacific Islander, and more than 1 race.

^c^ The missingness rate for NDI was 0.8%.

^d^ Office hours were defined as ED arrival times from 9 am to 5 pm, Monday through Friday.

^e^ Active treatment with a high-risk medication (chemotherapy, immunosuppressants, anticoagulants, and insulin or sulfonylureas) was defined as a medication dispensed within the 6 months prior to study ED visit.

^f^ The missingness rate for COPS2, a longitudinal comorbidity score based on 12 months of patient data,^41^ was 18.2%.

Certain clinical and visit characteristics were associated with risk of mistriage. Patients arriving during nonoffice hours were more likely to be both overtriaged and undertriaged, and patients arriving by ambulance were more likely to be undertriaged. The relative risk of undertriage was 30.3% (95% CI, 28.3%-32.4%) greater among patients using insulin or sulfonylureas, 22.4% (95% CI, 20.1%-24.4%) greater among patients with high comorbidity burdens, and 36.7% (95% CI, 30.5%-41.4%) greater among patients with a recent ICU admission. In sensitivity analyses, the same sociodemographic and clinical characteristics were associated with both undertriage and overtriage among patients with and without KPNC health plan membership, including sociodemographic characteristics, COPS2 scores or categories and imputed COPS2 scores, medication use, prior health care utilization, and visit time.

## Discussion

We developed a novel algorithm to assess mistriage that can be applied across large, automated data sets. In a multicenter cohort, mistriage occurred in almost one-third of encounters, and the sensitivity of ESI to identify a critically ill patient was only 65.9%. While ED triage is an inherently challenging process because of the need to predict patient interventions rapidly with limited information, our findings indicate that improvement of version 4 of the ESI was needed. Mistriage may have led to inadequate prioritization of limited resources to those who need them most urgently. This may lead to undertriage that delays treatment^28,29,30,31,32^ and a loss of operational efficiency in the case of overtriage.^24,33,35^ In adjusted analyses, we found sociodemographic disparities in triage accuracy, with higher rates of mistriage among young, Black, Hispanic, and male patients, those living in poorer neighborhoods, and those arriving during evenings, nights, and weekends. Last, we identified multiple high-risk clinical characteristics that could be used to improve triage predictions.

To the best of our knowledge, this is the largest and most robust analysis of the validity of ESI (version 4) that was used in over 70% of EDs across the US.^9^ Earlier studies, all prior to implementation of ESI version 5, had small sample sizes, used single outcome measures^15,44^ or heterogenous definitions of mistriage,^15,25,44^ or expressed expert opinion via case simulations or EHR review. These studies estimated that mistriage occurs in 12% to 45% of encounters using ESI, with estimates varying based on mistriage definition.^15,19,26,44^

Using EHR data to define mistriage, we estimate that just under one-third of ED encounters were mistriaged with ESI (version 4). Undertriage was much less common than overtriage. This may be because life-saving interventions are relatively rare and triage nurses may be more explicitly trained to identify sick patients. We found that one-third of patients who had a critical illness were assigned a middle- or low-acuity triage level (ESI III-V). Clinical examples of this included patients with acute strokes requiring thrombolytic therapy triaged as ESI IV and patients with septic shock requiring intubation and vasopressors triaged as ESI III. This low sensitivity of ESI to accurately identify critically ill patients may have led to delays in care and may contribute to increased morbidity and mortality.^28,29,30,32^

The clinical importance of the 3.3% of encounters that were undertriaged is likely variable. The Figure demonstrates that approximately two-thirds of cases of undertriage may have less clinical importance. Although beyond the scope of this analysis, we suspect there may still be some operational importance of underestimating resource needs by impacting where patients are seen (fast-track space vs main ED) and ED length of stay.

Almost 30% of patients were overtriaged, and we estimate the sensitivity of version 4 of the ESI for identifying low resource use and low-acuity illness among patients was only 50%. This poor differentiation of lower-risk patients may have biased physician downstream resource utilization^33^ and contributed to ED crowding by patients who might be better served in fast-track spaces. Prior studies^45,46^ have shown that interventions that efficiently separate out patients with low-acuity illness decrease ED length of stay and improve patient satisfaction without negatively affecting quality. Emergency department physicians can also vary in their resource utilization and admission decisions 2-fold.^47,48^ This variation, as well as a lower emphasis on training triage nurses to estimate resource use, may contribute to higher rates of overtriage compared with undertriage.

We found disparities in triage accuracy by race and ethnicity, sex, age, and socioeconomic status. Several studies demonstrate disparities in outcomes across multiple emergency conditions.^49,50,51,52^ Disparities in triage accuracy by patient attributes may contribute to disparate outcomes for time-sensitive conditions.^28,29,30,31,32^ This highlights another limitation of triage algorithms using subjective branch points and mirrors findings of disparities in triage accuracy by sociodemographic characteristics found in earlier studies.^53,54,55,56^ Exploring methods to limit disparities or using objective triage scores may help promote more equitable triage processes and patient outcomes. We found variation by patient arrival time and medical center, again highlighting the need to develop more data-driven triage processes to standardize care across time and place.

Our findings are designed to offer a novel characterization of the problem of mistriage and support future examination of patient outcomes and design of processes to improve triage accuracy, equity, and patient outcomes. Interventions to decrease undertriage may lead to more overtriage, and vice versa. A more granular understanding of the relative impacts of undertriage and overtriage on patient outcomes and resource utilization may allow systems to determine benchmarks for quality improvement and better understand acceptable levels of undertriage and overtriage.

Our novel measures of mistriage likely have broad applications. Because they rely on automated EHR data, other health systems may use these algorithms as a quality measure to assess the quality of ED nurse triage and for quality improvement purposes. Additionally, they could be developed in a quality measure used for public accountability. Quality improvement efforts should focus on reducing undertriage of critically ill patients and limiting disparities in triage accuracy. The ESI version 5 Implementation Handbook provides detailed explanations to more accurately categorize levels 1 and 2 patients, and a new section of the handbook addresses the role of racism, bias, and stigmas.^12^ Follow-up studies are needed to evaluate the impact of these updates on triage accuracy and equity.

We found that key patient history characteristics may be associated with acuity and resource needs, suggesting opportunities to improve prediction using EHR data. Triage clinicians have limited time and must make triage assignments with limited information. A flag for certain characteristics that often correlate with higher acuity or resource needs, such as outpatient sulfonylurea or insulin use (30% increased risk of undertriage) or recent ICU admission (36% increased risk of undertriage) could help limit mistriage and delays in care. We found use of dextrose 50% among patients assigned ESI III was a frequent driver of algorithm-determined undertriage. An ESI II assignment for these patients might allow for earlier evaluation and treatment, for example, with diet orders, glucose level checks, or appropriate medications.^57^

Previous studies have shown promise in using prediction models with EHR data to improve triage decisions. Levin et al^34^ found that an electronic triage system improved discrimination of patients with midacuity illness and increased sensitivity compared with ESI version 4 to identify patients needing hospital admission. Fenn et al^58^ used models that predict hospital and ICU admission and demonstrated high accuracy in prospective evaluation, although they have not yet been studied in real-time use.

### Limitations

This study has some limitations. We used version 4 of the ESI, which was in use by the included EDs at the time of this study. The subsequently released version 5 of ESI was not included in this study, and thus, these findings cannot be generalized to the currently available version of the ESI.

In addition, we were limited in developing mistriage measures that relied on readily available EHR data and that could be broadly applied to all conditions among adult patients. Our findings cannot be generalized to pediatric ED patients. Subjective elements such as distress, confusion, or pain, or psychosocial complexity are important measures of acuity and resource needs but challenging to capture. Although use of time stamps allowed us to consider the dynamic nature of patients’ conditions after ED triage, our measures cannot fully account for meaningful and unexpected clinical deterioration after triage.

The last 9 months of the study period occurred during the COVID-19 pandemic. In adjusted analyses, we found a lower risk of overtriage and a comparable rate of undertriage in 2020 compared with other study years. There were changes in ED volume, patient acuity, and operational protocols during the first 9 months of the pandemic, and we are not able to assess how these multiple changes may have impacted triage accuracy.

We conducted our study among a diverse population of patients across 21 community EDs. Close to 80% of encounters were by KPNC members, and the comprehensive EHR data for these patients allowed for more granular analysis. While rates of mistriage were higher among nonmembers, the association of key patient and visit characteristics with mistriage were similar among members and nonmembers, suggesting our findings are generalizable to other populations. Still, application of these algorithms in different settings to assess frequency of mistriage may be limited by availability of EHR data. Additionally, we did not assess patient outcomes in this study, and more research is needed to understand how mistriage may be associated with quality of care or resource decisions, the ideal balance of undertriage and overtriage, or how efforts to decrease undertriage might impact overtriage, patient outcomes, and operational flow.

## Conclusions

In this cohort study, we developed operational definitions to measure undertriage and overtriage to assess triage accuracy in a large, diverse adult population based on the use of version 4 of the ESI. Mistriage occurred in almost one-third of patients. There were disparities in triage accuracy by race and ethnicity, sex, age, neighborhood poverty level, ED arrival time, and ED location. In addition, we identified several high-risk clinical characteristics that could be used to improve accuracy of triage predictions. These findings highlight the need for more data-driven protocols to improve the safety, quality, and equity of ED triage. Future research should include assessments based on version 5 of the ESI, which was released after this study was completed.

## Notes

Supplement 1. **eFigure.** Study Cohort Assembly **eTable 1.** Intervention Levels **eTable 2.** Rates of Resource Utilization Among All Included Encounters and by Version 4 of the Emergency Severity Index (ESI) Assignment **eTable 3.** Rates of Level 1-4 Interventions Among Full Study Cohort and by Version 4 of the Emergency Severity Index (ESI) Assignment

Supplement 2. **Nonauthor Collaborators. **The Kaiser Permanente CREST (Clinical Research on Emergency Services & Treatments) Network

Supplement 3.
Data Sharing Statement

## References

[1] Herring AA, Johnson B, Ginde AA, . High-intensity emergency department visits increased in California, 2002-09. Health Aff (Millwood). 2013;32(10):1811-1819. doi:10.1377/hlthaff.2013.0397 24101073 PMC4109394

[2] Bernstein SL, Aronsky D, Duseja R, ; Society for Academic Emergency Medicine, Emergency Department Crowding Task Force. The effect of emergency department crowding on clinically oriented outcomes. Acad Emerg Med. 2009;16(1):1-10. doi:10.1111/j.1553-2712.2008.00295.x 19007346

[3] Pines JM, Griffey RT. What we have learned from a decade of ED crowding research. Acad Emerg Med. 2015;22(8):985-987. doi:10.1111/acem.12716 26194441

[4] Pines JM, Hollander JE. Emergency department crowding is associated with poor care for patients with severe pain. Ann Emerg Med. 2008;51(1):1-5. doi:10.1016/j.annemergmed.2007.07.008 17913299

[5] Intas G, Stergiannis P, Chalari E, Tsoumakas K, Fildissis G. The impact of ED boarding time, severity of illness, and discharge destination on outcomes of critically ill ED patients. Adv Emerg Nurs J. 2012;34(2):164-169. doi:10.1097/TME.0b013e318251515f 22561226

[6] Cowan RM, Trzeciak S. Clinical review: emergency department overcrowding and the potential impact on the critically ill. Crit Care. 2005;9(3):291-295. doi:10.1186/cc2981 15987383 PMC1175862

[7] Guttmann A, Schull MJ, Vermeulen MJ, Stukel TA. Association between waiting times and short term mortality and hospital admission after departure from emergency department: population based cohort study from Ontario, Canada. BMJ. 2011;342:d2983. doi:10.1136/bmj.d2983 21632665 PMC3106148

[8] Carter EJ, Pouch SM, Larson EL. The relationship between emergency department crowding and patient outcomes: a systematic review. J Nurs Scholarsh. 2014;46(2):106-115. doi:10.1111/jnu.12055 24354886 PMC4033834

[9] McHugh M, Tanabe P, McClelland M, Khare RK. More patients are triaged using the Emergency Severity Index than any other triage acuity system in the United States. Acad Emerg Med. 2012;19(1):106-109. doi:10.1111/j.1553-2712.2011.01240.x 22211429

[10] Wuerz RC, Milne LW, Eitel DR, Travers D, Gilboy N. Reliability and validity of a new five-level triage instrument. Acad Emerg Med. 2000;7(3):236-242. doi:10.1111/j.1553-2712.2000.tb01066.x 10730830

[11] Gilboy N, Tanabe T, Travers D, Rosenau AM. Emergency Severity Index (ESI): a triage tool for emergency department care, version 4. Implementation Handbook 2012 Edition. AHRQ Publication No. 12-0014. Agency for Healthcare Research and Quality; 2011.

[12] . Emergency Nurses Association. *Emergency Severity Index Handbook Fifth Edition*. 2023. Accessed May 24, 2024. https://californiaena.org/wp-content/uploads/2023/05/ESI-Handbook-5th-Edition-3-2023.pdf

[13] Gilboy N, Tanabe P, Travers DA. The Emergency Severity Index Version 4: changes to ESI level 1 and pediatric fever criteria. J Emerg Nurs. 2005;31(4):357-362. doi:10.1016/j.jen.2005.05.011 16126100

[14] Grossmann FF, Zumbrunn T, Frauchiger A, Delport K, Bingisser R, Nickel CH. At risk of undertriage? testing the performance and accuracy of the emergency severity index in older emergency department patients. Ann Emerg Med. 2012;60(3):317-325.e3. doi:10.1016/j.annemergmed.2011.12.013 22401951

[15] Hinson JS, Martinez DA, Cabral S, . Triage performance in emergency medicine: a systematic review. Ann Emerg Med. 2019;74(1):140-152. doi:10.1016/j.annemergmed.2018.09.022 30470513

[16] Platts-Mills TF, Travers D, Biese K, . Accuracy of the Emergency Severity Index triage instrument for identifying elder emergency department patients receiving an immediate life-saving intervention. Acad Emerg Med. 2010;17(3):238-243. doi:10.1111/j.1553-2712.2010.00670.x 20370755

[17] Mirhaghi A, Ebrahimi M. High risk criteria in level 2 may provide a source of disagreement in Emergency Severity Index. Bull Emerg Trauma. 2019;7(1):90-91. doi:10.29252/beat-070116 30719475 PMC6360001

[18] Mistry B, Balhara KS, Hinson JS, . Nursing perceptions of the Emergency Severity Index as a triage tool in the United Arab Emirates: a qualitative analysis. J Emerg Nurs. 2018;44(4):360-367. doi:10.1016/j.jen.2017.10.012 29167033

[19] Mistry B, Stewart De Ramirez S, Kelen G, . Accuracy and reliability of emergency department triage using the Emergency Severity Index: an international multicenter assessment. Ann Emerg Med. 2018;71(5):581-587.e3. doi:10.1016/j.annemergmed.2017.09.036 29174836

[20] Christ M, Grossmann F, Winter D, Bingisser R, Platz E. Modern triage in the emergency department. Dtsch Arztebl Int. 2010;107(50):892-898. 21246025 10.3238/arztebl.2010.0892 PMC3021905

[21] Martin A, Davidson CL, Panik A, Buckenmyer C, Delpais P, Ortiz M. An examination of ESI triage scoring accuracy in relationship to ED nursing attitudes and experience. J Emerg Nurs. 2014;40(5):461-468. doi:10.1016/j.jen.2013.09.009 24290530

[22] Ghafarypour-Jahrom M, Taghizadeh M, Heidari K, Derakhshanfar H. Validity and reliability of the Emergency Severity Index and Australasian triage system in pediatric emergency care of Mofid Children’s Hospital in Iran. Bull Emerg Trauma. 2018;6(4):329-333. doi:10.29252/beat-060410 30402522 PMC6215064

[23] Zachariasse JM, Kuiper JW, de Hoog M, Moll HA, van Veen M. Safety of the Manchester Triage System to detect critically ill children at the emergency department. J Pediatr. 2016;177:232-237.e1. doi:10.1016/j.jpeds.2016.06.068 27480197

[24] Hinson JS, Martinez DA, Schmitz PSK, . Accuracy of emergency department triage using the Emergency Severity Index and independent predictors of under-triage and over-triage in Brazil: a retrospective cohort analysis. Int J Emerg Med. 2018;11(1):3. doi:10.1186/s12245-017-0161-8 29335793 PMC5768578

[25] Lentz BA, Jenson A, Hinson JS, . Validity of ED: addressing heterogeneous definitions of over-triage and under-triage. Am J Emerg Med. 2017;35(7):1023-1025. doi:10.1016/j.ajem.2017.02.012 28188059

[26] Travers DA, Waller AE, Katznelson J, Agans R. Reliability and validity of the Emergency Severity Index for pediatric triage. Acad Emerg Med. 2009;16(9):843-849. doi:10.1111/j.1553-2712.2009.00494.x 19845551

[27] Baumann MR, Strout TD. Evaluation of the Emergency Severity Index (version 3) triage algorithm in pediatric patients. Acad Emerg Med. 2005;12(3):219-224. doi:10.1197/j.aem.2004.09.023 15741584

[28] Weiss SL, Fitzgerald JC, Balamuth F, . Delayed antimicrobial therapy increases mortality and organ dysfunction duration in pediatric sepsis. Crit Care Med. 2014;42(11):2409-2417. doi:10.1097/CCM.0000000000000509 25148597 PMC4213742

[29] Furnival RA, Woodward GA, Schunk JE. Delayed diagnosis of injury in pediatric trauma. Pediatrics. 1996;98(1):56-62. doi:10.1542/peds.98.1.56 8668413

[30] Wang TY, Nallamothu BK, Krumholz HM, . Association of door-in to door-out time with reperfusion delays and outcomes among patients transferred for primary percutaneous coronary intervention. JAMA. 2011;305(24):2540-2547. doi:10.1001/jama.2011.862 21693742

[31] Haas B, Gomez D, Zagorski B, Stukel TA, Rubenfeld GD, Nathens AB. Survival of the fittest: the hidden cost of undertriage of major trauma. J Am Coll Surg. 2010;211(6):804-811. doi:10.1016/j.jamcollsurg.2010.08.014 21036070

[32] Yurkova I, Wolf L. Under-triage as a significant factor affecting transfer time between the emergency department and the intensive care unit. J Emerg Nurs. 2011;37(5):491-496. doi:10.1016/j.jen.2011.01.016 21549418

[33] Calder LA, Forster AJ, Stiell IG, . Mapping out the emergency department disposition decision for high-acuity patients. Ann Emerg Med. 2012;60(5):567-576.e4. doi:10.1016/j.annemergmed.2012.04.013 22699018

[34] Levin S, Toerper M, Hamrock E, . Machine-learning–based electronic triage more accurately differentiates patients with respect to clinical outcomes compared with the Emergency Severity Index. Ann Emerg Med. 2018;71(5):565-574.e2. doi:10.1016/j.annemergmed.2017.08.005 28888332

[35] Chmielewski N, Moretz J. ESI triage distribution in US emergency departments. Adv Emerg Nurs J. 2022;44(1):46-53. doi:10.1097/TME.0000000000000390 35089282

[36] Fernandes M, Vieira SM, Leite F, Palos C, Finkelstein S, Sousa JMC. Clinical decision support systems for triage in the emergency department using intelligent systems: a review. Artif Intell Med. 2020;102:101762. doi:10.1016/j.artmed.2019.101762 31980099

[37] Farrohknia N, Castrén M, Ehrenberg A, . Emergency department triage scales and their components: a systematic review of the scientific evidence. Scand J Trauma Resusc Emerg Med. 2011;19:42. doi:10.1186/1757-7241-19-42 21718476 PMC3150303

[38] Krieger N. Overcoming the absence of socioeconomic data in medical records: validation and application of a census-based methodology. Am J Public Health. 1992;82(5):703-710. doi:10.2105/AJPH.82.5.703 1566949 PMC1694121

[39] Gordon N, Lin T. The Kaiser Permanente Northern California Adult Member Health Survey. Perm J. 2016;20(4):15-225. doi:10.7812/TPP/15-225 27548806 PMC5101088

[40] Hasson F, Keeney S, McKenna H. Research guidelines for the Delphi survey technique. J Adv Nurs. 2000;32(4):1008-1015. doi:10.1046/j.1365-2648.2000.t01-1-01567.x 11095242

[41] Escobar GJ, Ragins A, Scheirer P, Liu V, Robles J, Kipnis P. Nonelective rehospitalizations and postdischarge mortality: predictive models suitable for use in real time. Med Care. 2015;53(11):916-923. doi:10.1097/MLR.0000000000000435 26465120 PMC4605276

[42] Ramsey PH, Ramsey PP. Power of pairwise comparisons in the equal variance and unequal sample size case. Br J Math Stat Psychol. 2008;61(pt 1):115-131. doi:10.1348/000711006X153051 18482478

[43] Venkatesh AK, Janke AT, Shu-Xia L, . Emergency department utilization for emergency conditions during COVID-19. Ann Emerg Med. 2021;78(1):84-91. doi:10.1016/j.annemergmed.2021.01.011 33840512 PMC7805390

[44] Zachariasse JM, van der Hagen V, Seiger N, Mackway-Jones K, van Veen M, Moll HA. Performance of triage systems in emergency care: a systematic review and meta-analysis. BMJ Open. 2019;9(5):e026471. doi:10.1136/bmjopen-2018-026471 31142524 PMC6549628

[45] Wiler JL, Ozkaynak M, Bookman K, . Implementation of a front-end split-flow model to promote performance in an urban academic emergency department. Jt Comm J Qual Patient Saf. 2016;42(6):271-280. doi:10.1016/S1553-7250(16)42036-2 27184243

[46] Hwang CE, Lipman GS, Kane M. Effect of an emergency department fast track on Press-Ganey patient satisfaction scores. West J Emerg Med. 2015;16(1):34-38. doi:10.5811/westjem.2014.11.21768 25671005 PMC4307722

[47] Tavarez MM, Ayers B, Jeong JH, Coombs CM, Thompson A, Hickey RW. Practice variation and effects of e-mail–only performance feedback on resource use in the emergency department. Acad Emerg Med. 2017;24(8):948-956. doi:10.1111/acem.13211 28470786

[48] Jain S, Elon LK, Johnson BA, Frank G, Deguzman M. Physician practice variation in the pediatric emergency department and its impact on resource use and quality of care. Pediatr Emerg Care. 2010;26(12):902-908. doi:10.1097/PEC.0b013e3181fe9108 21088636

[49] Bazarian JJ, Pope C, McClung J, Cheng YT, Flesher W. Ethnic and racial disparities in emergency department care for mild traumatic brain injury. Acad Emerg Med. 2003;10(11):1209-1217. doi:10.1197/S1069-6563(03)00491-3 14597497

[50] Goyal MK, Hayes KL, Mollen CJ. Racial disparities in testing for sexually transmitted infections in the emergency department. Acad Emerg Med. 2012;19(5):604-607. doi:10.1111/j.1553-2712.2012.01338.x 22594368

[51] Carreras Tartak JA, Brisbon N, Wilkie S, . Racial and ethnic disparities in emergency department restraint use: a multicenter retrospective analysis. Acad Emerg Med. 2021;28(9):957-965. doi:10.1111/acem.14327 34533261

[52] Lee P, Le Saux M, Siegel R, . Racial and ethnic disparities in the management of acute pain in US emergency departments: meta-analysis and systematic review. Am J Emerg Med. 2019;37(9):1770-1777. doi:10.1016/j.ajem.2019.06.014 31186154

[53] Dennis JA. Racial/ethnic disparities in triage scores among pediatric emergency department fever patients. Pediatr Emerg Care. 2021;37(12):e1457-e1461. doi:10.1097/PEC.0000000000002072 32150002

[54] Zook HG, Kharbanda AB, Flood A, Harmon B, Puumala SE, Payne NR. Racial differences in pediatric emergency department triage scores. J Emerg Med. 2016;50(5):720-727. doi:10.1016/j.jemermed.2015.02.056 26899520 PMC4851931

[55] Schrader CD, Lewis LM. Racial disparity in emergency department triage. J Emerg Med. 2013;44(2):511-518. doi:10.1016/j.jemermed.2012.05.010 22818646

[56] Vigil JM, Alcock J, Coulombe P, . Ethnic disparities in Emergency Severity Index scores among US Veteran’s Affairs emergency department patients. PLoS One. 2015;10(5):e0126792. doi:10.1371/journal.pone.0126792 26024515 PMC4449190

[57] Moore C, Woollard M. Dextrose 10% or 50% in the treatment of hypoglycaemia out of hospital? a randomised controlled trial. Emerg Med J. 2005;22(7):512-515. doi:10.1136/emj.2004.020693 15983093 PMC1726850

[58] Fenn A, Davis C, Buckland DM, . Development and validation of machine learning models to predict admission from emergency department to inpatient and intensive care units. Ann Emerg Med. 2021;78(2):290-302. doi:10.1016/j.annemergmed.2021.02.029 33972128

