# Clarification of Version Index Used in Study

**DOI:** 10.1001/jamanetworkopen.2024.23536

**Published:** 2024-06-13

In the Original Investigation titled “Evaluation of Version 4 of the Emergency Severity Index in US Emergency Departments for the Rate of Mistriage,”^1^ published March 17, 2023, clarifications were needed to indicate that this study was based on version 4 of the Emergency Severity Index (ESI), to indicate the provenance of the ESI and its versions, and to provide references to support these clarifications. In addition, Figure 1, which presented the ESI triage algorithm, has been removed because permission to republish it had not been obtained. The article has been corrected.
