# Supplementary material for: Evaluation of Version 4 of the Emergency Severity Index in US Emergency Departments for the Rate of Mistriage
Source: JAMA Netw Open. 2023 Mar 17;6(3):e233404. doi: 10.1001/jamanetworkopen.2023.3404 (PMC10024207; doi:10.1001/jamanetworkopen.2023.3404)
Supplement: Supplement 1. — eFigure. Study Cohort Assembly eTable 1. Intervention Levels eTable 2. Rates of Resource Utilization Among All Included Encounters and by Version 4 of the Emergency Severity Index (ESI) Assignment eTable 3. Rates of Level 1-4 Interventions Among Full Study Cohort and by Version 4 of the Emergency Severity Index (ESI) Assignment [file jamanetwopen-e233404-s001.pdf]

Supplementary Online Content

Sax DR, Warton EM, Mark DG, et al; Kaiser Permanente CREST (Clinical Research on Emergency Services & Treatments) Network. Evaluation of the Emergency Severity Index in US emergency departments for the rate of mistriage. *JAMA Netw Open*. 2023;6(3):e233404. doi:10.1001/jamanetworkopen.2023.3404

eFigure. Study Cohort Assembly
eTable 1. Intervention Levels
eTable 2. Rates of Resource Utilization Among All Included Encounters and by Version 4 of the Emergency Severity Index (ESI) Assignment
eTable 3. Rates of Level 1-4 Interventions Among Full Study Cohort and by Version 4 of the Emergency Severity Index (ESI) Assignment

This supplementary material has been provided by the authors to give readers additional information about their work.

© 2023 Sax DR et al. *JAMA Network Open*.

eFigure. Study Cohort Assembly

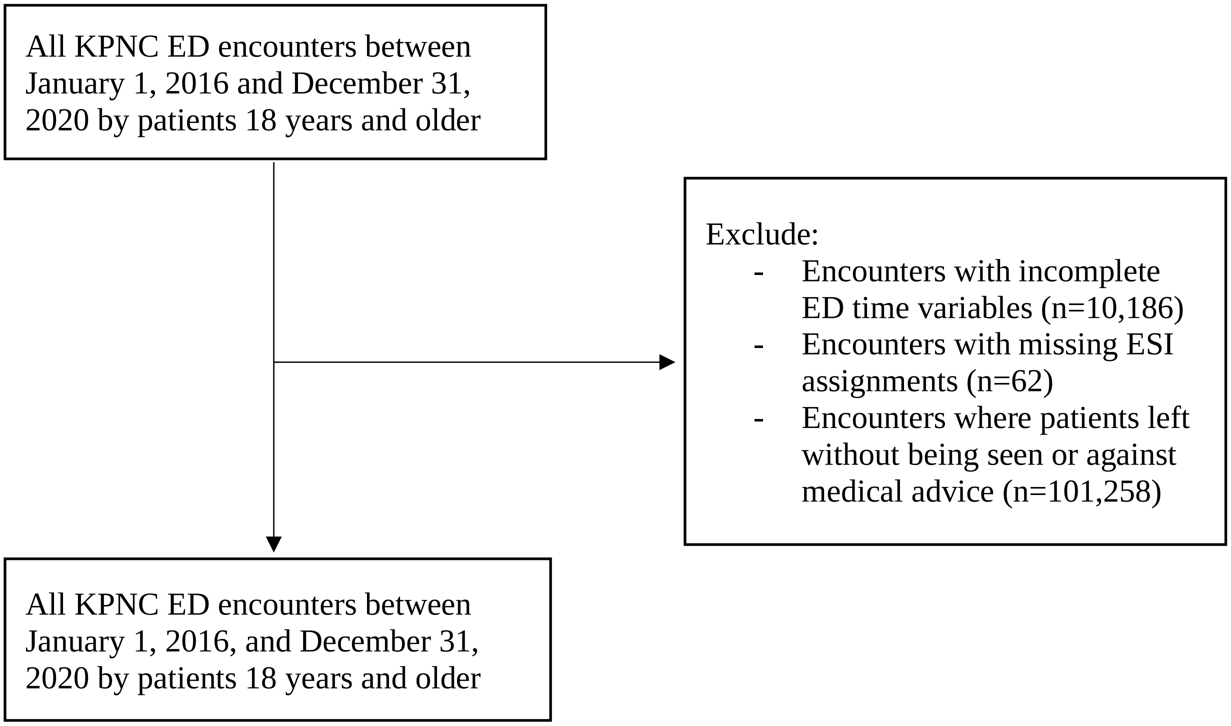

Abbreviations: ED, emergency department; KPNC, Kaiser Permanente Northern California

© 2023 Sax DR et al. *JAMA Network Open*.

eTable 1. Intervention Levels

**Intervention level**	**Description**
1	Lifesaving intervention within 1 h of arrival in ED:<br>• Invasive ventilation<br>• Tier I critical medications used: epinephrine, norepinephrine, vasopressin, dopamine, dobutamine, phenylephrine, isoproterenol, atropine, tenecteplase, alteplase<br>• Admission to catheterization suite, operating room, or ICU or transfer to another hospital<br>• Blood transfusion: fresh frozen plasma, platelets, prothrombin complex concentrate, or ≥1 unit packed red blood cells<br>• Death in the ED
2	Any of these specific level 1 interventions beyond first hour:<br>• Invasive ventilation, tier 1 critical medication, admission to catheterization suite, massive transfusion protocol, >2 units blood, or death in the ED<br>• Any tier II critical medication used (at any time): nicardipine, sodium nitroprusside, esmolol, milrinone, labetalol drip, nitroglycerin drip, dextrose 50%, naloxone, calcium gluconate or calcium chloride, sodium bicarbonate<br>• Parenteral psychotropic medication administered within 120 min: haloperidol lactate (≥5 mg if patient aged <65 y, ≥2 mg if aged ≥65 y), lorazepam (≥2 mg), olanzapine (≥10 mg), or ziprasidone mesylate (≥10 mg)<br>• Noninvasive ventilation<br>• Intraosseous line placed
3	• Admission to ICU or operating room or transfer to another hospital beyond first hour<br>• Critical procedures: central line, arterial line, paracentesis, thoracentesis, tube thoracostomy, and lumbar puncture<br>• Tier III critical medication used (at any time): parenteral procainamide,

© 2023 Sax DR et al. *JAMA Network Open*.

		amiodarone, ibutilide, heparin, insulin drip, continuous albuterol<br>• No level 1 or 2 intervention
4		• Tier IV medications used (at any time): parenteral etomidate, ketamine, propofol, metoprolol, diltiazem, adenosine, digoxin, hydralazine, labetalol, or sublingual or transdermal nitroglycerin<br>• 1-2 units packed red blood cell or any other blood product transfusion beyond first hour<br>• No level 1, 2, or 3 intervention

Abbreviations: ED, emergency department; ICU, intensive care unit; mg, milligrams; y, years

© 2023 Sax DR et al. *JAMA Network Open*.

eTable 2. Rates of Resource Utilization Among All Included Encounters and by Version 4 of the Emergency Severity Index (ESI) Assignment

Number of types of resources used	All patients n=5,315,176 (%)	ESI I n=33,491 (%)	ESI II n=929,555 (%)	ESI III n=3,262,047 (%)	ESI IV n=1,046,806 (%)	ESI V n=43,277 (%)
0	949,401 (17.9)	127 (0.38)	22,769 (2.45)	351,189 (10.8)	538,960 (51.5)	36,356 (84.0)
1	1,027,855 (19.3)	1551 (4.6)	53,662 (5.8)	558,713 (17.1)	407,864 (39.0)	6,065 (14.0)
2+	3,337,920 (62.8)	31,813 (95.0)	853,124 (91.8)	2,352,145 (72)	99,982 (9.6)	856 (2.0)

Notes: Resource use was defined as it is in version 4 of the Emergency Severity Index, and each different type of resource is counted as a resource, not the individual tests or imaging studies. We only included resources that were measured electronically. Resource types include laboratory analysis, electrocardiograms, radiology exams (X-ray, CT scan, MRI, and ultrasound), intravenous fluids, and intravenous or intramuscular medications. ESI I cases should represent emergent, life-threatening conditions, whereas ESI V cases should represent low acuity, low resource-need patients. Percentages in each cell are column percentages.

eTable 3. Rates of Level 1-4 Interventions Among Full Study Cohort and by Version 4 of the Emergency Severity Index (ESI) Assignment

Intervention Level	All patients n=5,315,176 (%)	ESI I n=33,491 (%)	ESI II n=929,555 (%)	ESI III n=3,262,047 (%)	ESI IV n=1,046,806 (%)	ESI V n=43,277 (%)
Level 1 (most severe)	28,383 (0.5)	11,091 (33.1)	13,278 (1.43)	3,809 (0.1)	196 (0.02)	9 (0.02)
Level 2	136,207 (2.6)	10,329 (30.8)	73,715 (7.9)	51,792 (1.6)	359 (0.03)	12 (0.03)
Level 3	189,553 (3.6)	2,794 (8.3)	71,862 (7.7)	113,893 (3.5)	1,004 (0.1)	0 (0.0)
Level 4 (least severe)	161,193 (3.0)	1,403 (4.2)	77,606 (8.4)	81,569 (2.5)	613 (0.06)	2 (0.0)
Hospital admission	673,659 (12.7)	21,069 (62.9)	283,048 (30.5)	367,431 (11.3)	2,085 (0.2)	26 (0.06)
No Level 1-4 interventions	4,799,840 (90.3)	7,824 (23.5)	693,094 (74.6)	3,010,984 (92.3)	1,044,634 (99.8)	43,254 (100)

Notes: Please see Table 1 above for Level 1-4 intervention definitions. ESI I cases should represent emergent, life-threatening conditions, whereas ESI V cases should represent low acuity, low resource-need patients. Percentages in each cell are column percentages.

© 2023 Sax DR et al. *JAMA Network Open*.
