# Supplementary material for: Evaluation of Version 4 of the Emergency Severity Index in US Emergency Departments for the Rate of Mistriage
Source: JAMA Netw Open. 2023 Mar 17;6(3):e233404. doi: 10.1001/jamanetworkopen.2023.3404 (PMC10024207; doi:10.1001/jamanetworkopen.2023.3404)
Supplement: Supplement 2. — Nonauthor Collaborators. The Kaiser Permanente CREST (Clinical Research on Emergency Services & Treatments) Network [file jamanetwopen-e233404-s002.pdf]

Supplemental Online Content: Nonauthor Collaborators
*First name, last name, and suffix (if applicable) are required and will appear in PubMed.

***Group Name(s): Kaiser Permanente CREST (Clinical Research on Emergency Services & Treatments) Network**							
***First Name and Middle Initial(s)**	***Last Name**	***Suffix (eg, Jr, III)**	Academic Degrees	Institution	Location (city, state/province, country)	Role or Contribution, eg, chair, principal investigator	Group (if more than 1 Group listed in the byline) and/or Subgroup (eg, Steering Committee)
Adina S	Rauchwerger		MPH	Kaiser Permanente Northern California Division of Research	Oakland, CA	Project Manager	
Jennifer Y	Zhang		BS	Kaiser Permanente Northern California Division of Research	Oakland, CA	Research Assistant	
