# Supplementary material for: Evaluation of Version 4 of the Emergency Severity Index in US Emergency Departments for the Rate of Mistriage
Source: JAMA Netw Open. 2023 Mar 17;6(3):e233404. doi: 10.1001/jamanetworkopen.2023.3404 (PMC10024207; doi:10.1001/jamanetworkopen.2023.3404)
Supplement: Supplement 3. — Data Sharing Statement [file jamanetwopen-e233404-s003.pdf]

Data Sharing Statement

Sax. Evaluation of Version 4 of the Emergency Severity Index in US Emergency Departments for the Rate of Mistriage. *JAMA Netw Open*. Published March 17, 2023. doi:10.1001/jamanetworkopen.2023.3404

Data
Data available: No

Additional Information
Explanation for why data not available: We will share aggregate data and define all predictor variables and outcomes, but we are not able to share individual patient data.
